# Analysis of Individual $\dot{V}$O_2max_ Responses during a Cardiopulmonary Exercise Test and the Verification Phase in Physically Active Women

**DOI:** 10.3390/jfmk8030124

**Published:** 2023-08-31

**Authors:** Pasquale J. Succi, Brian Benitez, Minyoung Kwak, Haley C. Bergstrom

**Affiliations:** Department of Kinesiology and Health Promotion, University of Kentucky, Lexington, KY 40536, USA; pj.succi@uky.edu (P.J.S.); bbe241@uky.edu (B.B.); mkw223@uky.edu (M.K.)

**Keywords:** exercise test, women, verification phase, oxygen consumption

## Abstract

This study aimed to investigate the test–retest reliability, mean, and individual responses in the measurement of maximal oxygen consumption ($\dot{V}$O_2max_) during a cardiopulmonary exercise test (CPET) and the verification phase during cycle ergometry in women. Nine women (22 ± 2 yrs, 166.0 ± 4.5 cm, 58.6 ± 7.7 kg) completed a CPET, passively rested for 5 min, and then completed a verification phase at 90% of peak power output to determine the highest $\dot{V}$O_2_ from the CPET ($\dot{V}$O_2CPET_) and verification phase ($\dot{V}$O_2verification_) on 2 separate days. Analyses included a two-way repeated measures ANOVA, intraclass correlation coefficients (ICC_2,1_), standard errors of the measurement (SEM), minimal differences (MD), and coefficients of variation (CoV). There was no test (test 1 versus test 2) × method (CPET vs. verification phase) interaction (*p* = 0.896) and no main effect for method (*p* = 0.459). However, test 1 (39.2 mL·kg^−1^·min^−1^) was significantly higher than test 2 (38.3 mL·kg^−1^·min^−1^) (*p* = 0.043). The $\dot{V}$O_2CPET_ (ICC = 0.984; CoV = 1.98%; SEM = 0.77 mL·kg^−1^·min^−1^; MD = 2.14 mL·kg^−1^·min^−1^) and $\dot{V}$O_2verification_ (ICC = 0.964; CoV = 3.30%; SEM = 1.27 mL·kg^−1^·min^−1^; MD = 3.52 mL·kg^−1^·min^−1^) demonstrated “excellent” reliability. Two subjects demonstrated a test 1 $\dot{V}$O_2CPET_ that exceeded the test 2 $\dot{V}$O_2CPET_, and one subject demonstrated a test 1 $\dot{V}$O_2verification_ that exceeded the test 2 $\dot{V}$O_2verification_ by more than the respective CPET and verification phase MD. One subject demonstrated a $\dot{V}$O_2CPET_ that exceeded the $\dot{V}$O_2verification_, and one subject demonstrated a $\dot{V}$O_2verification_ that exceeded the $\dot{V}$O_2CPET_ by more than the MD. These results demonstrate the importance of examining the individual responses in the measurement of the $\dot{V}$O_2max_ and suggest that the MD may be a useful threshold to quantify real individual changes in $\dot{V}$O_2_.

## 1. Introduction

The examination of individual responses to an exercise or nutritional intervention has gained increasing interest with the development of individualized exercise prescription, medicine, and genetic testing [1,2,3,4]. Despite this increased focus on individual responses, most primary research continues to base conclusions on group or mean effects. For example, training interventions that have examined changes in the volume of maximal oxygen consumption ($\dot{V}$O_2max_) utilized the mean response alone as justification for or against the efficacy of the given training protocol, despite high variability in the individual responses [4]. Therefore, the main effect and overall conclusion about the efficacy of the intervention may be driven by a few individuals that demonstrated exaggerated responses [5]. The misstep of drawing conclusions based primarily on the mean responses has also been observed in the examination of the methodologies used for the determination of the primary outcomes such as the $\dot{V}$O_2max_. Specifically, the call to perform a verification phase upon completion of an initial cardiopulmonary exercise test (CPET) to verify that the $\dot{V}$O_2max_ was achieved has been made primarily based on the mean responses [6,7,8,9,10,11]. However, the determination of the $\dot{V}$O_2max_ should be made using individual thresholds, since the mean responses do not identify those who have or have not attained a ‘true’ $\dot{V}$O_2max_. The few studies that have examined the individual responses have used a threshold based solely on the measurement error of the respective metabolic analyzer used, corresponding to a 2–3% difference, to determine significance among the individual responses [4,7,12,13,14]. Thus, there is a need for a method to compare individual responses that is based on the combined biological variability in addition to the error of the measurement being examined.

Previous work [15,16,17] has called for a test–retest approach to the quantification of individual responses. In particular, Weir [17] advocated for the use of the minimal difference (MD) to be considered real, which represents a 95% confidence interval around the standard error of the measurement (SEM), as a more statistically grounded threshold to determine ‘real’ individual differences test–retest. The MD is derived from test and subsequent retest values of the measure of interest, each with a true component and an error component [17]. By using the test and retest values, the MD thereby contains the error from the biological variability in addition to the measurement error of the given test [17]. Thus, the MD can be used to examine whether an individual difference from one test to another is the result of a ‘real’ difference, or if it is just the result of the day-to-day variability associated with the measure [17]. By quantifying the MD in a given population for the measurement of a primary outcome, such as the $\dot{V}$O_2max_, future researchers may be able to use the MD as a threshold to examine individual differences during a training or interventional study.

The measurement of the $\dot{V}$O_2max_ is a prevalent primary outcome in the examination of endurance exercise; yet, debate exists surrounding its measurement [18]. Traditional definitions of the $\dot{V}$O_2max_ use the presence of a plateau in the $\dot{V}$O_2_ (<150 mL·min^−1^) with increasing work rate as the primary criterion or secondary criteria, such as the attainment of a percentage of the age-predicted maximal heart rate (HR), a respiratory exchange ratio (RER) of 1.1 or greater, and a rating of perceived exertion (RPE) greater than or equal to 17 [18,19,20,21]. These criteria have come under criticism [18] due to the low incidence of a plateau in $\dot{V}$O_2_, and the inability of the secondary criteria to distinguish between the individual variation in responses for those who truly did attain a $\dot{V}$O_2_ and those who did not [18,21,22]. To address these criticisms, there has been an increased call to perform a verification phase upon the completion of a CPET to verify the attainment of the $\dot{V}$O_2max_ [18]. However, there is no consensus for a universal methodology for the administration of a verification phase to confirm the attainment of $\dot{V}$O_2max_. A recent review and meta-analysis with 54 studies found no difference between the highest $\dot{V}$O_2max_ attained in the CPET and that from the verification phase [23]. While the authors concluded the verification phase appears to be a robust procedure to confirm a ‘true’ $\dot{V}$O_2max_ has been attained, they also questioned its necessity in all populations based on the lack of mean differences between the $\dot{V}$O_2max_ from the CPET ($\dot{V}$O_2CPET_) and the verification phase ($\dot{V}$O_2verification_). The purpose of a verification phase is to, on an individual basis, examine the $\dot{V}$O_2_ responses to verify the individual attainment of the $\dot{V}$O_2max_ [24]. Thus, to fully examine the necessity of the verification phase in the measurement of $\dot{V}$O_2max_, the individual $\dot{V}$O_2_ responses should be examined during the CPET and verification phase.

Similar to the lack of consensus on the verification phase methodology, there is a lack of consensus on the specific magnitude of the difference between the $\dot{V}$O_2CPET_ and $\dot{V}$O_2verification_ needed to detect a real change or indicate whether the result is a consequence of measurement error or biological variability [17]. To compound this issue, the specific number of subjects in a given sample that demonstrate a difference in the $\dot{V}$O_2_ responses is often not reported [10,11]. Previous works that have attempted to examine individual differences have used a 2–3% threshold to define an individual difference as real [4,7,12,14]. Using this threshold, Weatherwax et al. [4] demonstrated 2.6% of subjects (4 out of 156 tests) demonstrated a 3% difference between the $\dot{V}$O_2CPET_ and $\dot{V}$O_2verification_. Other more recent work [25] has extrapolated the $\dot{V}$O_2_ versus work rate relationship to predict the $\dot{V}$O_2verification_ and used the predicted value as a method to confirm the individual attainment of the $\dot{V}$O_2max_ following the recommendation of Midgley et al. [26]. These authors [25] demonstrated that the $\dot{V}$O_2verification_ was on average 5% lower than the $\dot{V}$O_2CPET_, but that on an individual basis, the $\dot{V}$O_2max_ was ‘confirmed’ in all participants since the difference between the predicted and actual $\dot{V}$O_2max_ was less than half the regression slope [25,26]. While this method represents an improvement upon the 3% threshold, this may not serve as a threshold for future studies to use to examine individual responses. Thus, the need still exists for a threshold that encapsulates the biological variability and the error associated with the measurement that can be potentially used as a threshold for future studies.

Previous work [27,28,29] has used the MD to examine the individual differences in the $\dot{V}$O_2CPET_ and $\dot{V}$O_2verification_ in treadmill running in men and women and in cycle ergometry in men. These works, in addition to others [4,25,30], demonstrated that young healthy subjects accustomed to exhaustive exercise seldom demonstrate differences between the $\dot{V}$O_2CPET_ and $\dot{V}$O_2verification_, and in some cases, they demonstrate no differences at all. The lack of individual subjects who demonstrate $\dot{V}$O_2verification_ values that are either equivalent or are significantly less than the $\dot{V}$O_2CPET_ [4,25,27,28,29] support previous work [23] that has questioned the need for the verification phase in measuring the $\dot{V}$O_2max_ in all populations and settings. The additional benefit of the quantification of the MD in specific populations and in different modalities is that future works, such as training or nutritional interventions, are able to use the MD as a threshold by which individual responses can be examined without the need to perform verification phases. However, no study has quantified the MD during cycle ergometry in women. Thus, the purpose of this study was to use a test–retest approach to examine the reliability, mean, and individual differences between the $\dot{V}$O_2CPET_ and $\dot{V}$O_2verification_ in healthy, recreationally trained, and well-motivated women during cycle ergometry. Based on previous examinations of the verification phase using similar methodologies [27,28,29], it was hypothesized that (1) both the $\dot{V}$O_2CPET_ and $\dot{V}$O_2verification_ would have ‘excellent’ test–retest reliabilities; (2) there would be no mean differences between the $\dot{V}$O_2CPET_ and $\dot{V}$O_2verification_; (3) no individual would exceed the MD between the test and retest difference for the $\dot{V}$O_2CPET_ or $\dot{V}$O_2verification_; and (4) based on the previously reported incidence of individual differences [4,13], two or fewer subjects would exhibit a difference between the $\dot{V}$O_2CPET_ and $\dot{V}$O_2verification_ that exceeded the MD.

## 2. Materials and Methods

### 2.1. Experimental Approach

This study used a test–retest design to determine the reliability and validity of the determination of $\dot{V}$O_2max_ with a verification phase. The study consisted of 3 visits total with each visit being separated by at least 48 h. The first visit consisted of a familiarization trial where subjects performed a cardiopulmonary exercise test (CPET) followed by a verification phase on an electronically braked cycle ergometer to familiarize themselves with the protocol and the effort required to determine the $\dot{V}$O_2CPET_ and $\dot{V}$O_2verification_. The second and third visits followed the same procedures as the familiarization visit and were used to determine the mean and individual differences in the measurement of the $\dot{V}$O_2CPET_ and the $\dot{V}$O_2verification_.

### 2.2. Subjects

Ten moderately trained recreationally active women were recruited from university students and from the general public in the surrounding area. One subject withdrew due to scheduling conflicts. Therefore, 9 women were included in the analyses (mean ± SD, 22 ± 2 years, 166.0 ± 4.5 cm, 58.6 ± 7.7 kg). The subjects’ physical activities included a combination of running (n = 7), cycling (n = 2), resistance training (n = 5), and yoga (n = 2). Individuals were eligible for inclusion if they had been endurance training 30 min a day, 5 days a week, for the past 6 months and had no known cardiovascular, metabolic, or musculoskeletal diseases or disorders. The subjects were asked to maintain their current level of physical activity, but to abstain from high intensity exercise at least 24 h prior to their testing session and abstain from caffeine consumption 4 h before their testing session. All subjects completed a health history form and signed a written informed consent document approved by the University Institutional Review Board for Human Subjects (IRB#64999) prior to beginning the study.

### 2.3. Graded Exercise Test with Verification Phase

Each subject performed 3 CPET’s on a calibrated cycle ergometer (Lode, Corival, Groningen, The Netherlands) on different days each separated by at least 48 h. The first visit served as a familiarization trial so that subjects understood the effort required for each visit. The second and third trials were used for the test–retest determination of the $\dot{V}$O_2CPET_ and $\dot{V}$O_2verification_. Each subject was fitted with a nose clip, mouthpiece mounted to a headset (Hans Rudolph 2700 breathing valve, Kansas City, MO, USA), and heart rate monitor (Polar Heart Watch system, Polar Electro Inc., Lake Success, NY, USA) during all visits. Expired gas samples were collected and analyzed using a calibrated TrueMax 2400 metabolic cart (Parvo Medics, Sandy, UT, USA). Prior to testing, the gas analyzers were calibrated to room air and gases of known concentrations, and the flow meter was calibrated using a 3L syringe. The oxygen ($\dot{V}$O_2_) and carbon dioxide ($\dot{V}$CO_2_) parameters were expressed as 20 s averages [31]. Each subject performed a 4-min warmup at 50 W at 70 rev·min^−1^ cadence, followed by one minute of passive rest. The CPET started at 50 W, and the power output was increased 30 W every two minutes until the subjects could no longer maintain the 70 rev·min^−1^ cadence despite strong verbal encouragement. After the subject signaled for exhaustion, the subject was given 5 min of passive recovery, then the power output was increased to 90% of their peak power from the CPET. This intensity was maintained until the subject could no longer maintain the 70 rev·min^−1^ cadence despite strong verbal encouragement. The protocol for the current study was based on the work of Sawyer et al. [11], which indicated that a 90% verification phase was the ideal intensity to elicit the highest $\dot{V}$O_2verification_ values compared to 80, 100, and 105% of peak power in moderately trained individuals. The greater $\dot{V}$O_2_ response at 90% peak power, relative to the other submaximal, maximal, or supramaximal intensities, likely resulted because there was sufficient time for the development of the $\dot{V}$O_2_ slow component phenomenon causing the $\dot{V}$O_2_ to increase to the $\dot{V}$O_2max_ [32]. The $\dot{V}$O_2CPET_ and $\dot{V}$O_2verificaiton_ were defined as the highest 20 s $\dot{V}$O_2_ value obtained from the step protocol and the verification phase, respectively. The rating of perceived exertion (RPE) was recorded using the Borg 6–20 scale at the end of each stage during the CPET and after each minute during the verification phase [33]. The respiratory exchange ratio (RER) was defined as the highest 20 s value obtained from the step protocol and verification phase, respectively. 

### 2.4. Statistical Analyses

Separate, 2 (Test [Test 1 vs. Test 2]) × 2 (Method [CPET vs Verification]) repeated measures analyses of variance (ANOVA) were used to examine the interaction and main effects for the mean responses for the highest $\dot{V}$O_2_ demonstrated from the CPET and verification phase ($\dot{V}$O_2CPET_ and $\dot{V}$O_2verification_), as well as for the time to exhaustion (T_Lim_), heart rate (HR), respiratory exchange ratio (RER), rating of perceived exertion (RPE), and power output for the CPET and the verification phase with appropriate follow-up pairwise comparisons. The test–retest reliability of each variable was calculated using an intraclass correlation coefficient (ICC, relative reliability) (2,1) model [17,34,35,36] using the equation:

(1) $${ICC}_{2,1}=\frac{{MS}_{S}-{MS}_{E}}{{MS}_{S}+\left(k-1\right){MS}_{E}+(\frac{k({MS}_{T}-{MS}_{E}}{n})},$$

where the MS_S_ is the mean square error of the between-subjects effects, MS_E_ is the mean square error of the within-subjects effects, and MS_T_ is the mean square factor of the within-subjects effects from separate one-way repeated measures ANOVA for each method (CPET, verification phase). Additionally, k is the number of tests (k = 2), and n represents the sample size. A 2,1 ICC model was selected so that the ICC values could be generalized to outside testers [17,34,35,36]. The ICC values were classified as “excellent” (0.80–1.0), “good” (0.60–0.80), or “poor” (<0.60) [37]. A 95% confidence interval (CI) was calculated around each ICC value to confirm the rejection of the null hypothesis that each ICC was statistically different from zero. The standard error of the measurement (SEM, absolute reliability) was calculated using the equation:

(2) $$SEM=\sqrt{{MS}_{E}}.$$

Additionally, the minimal difference to be considered real (MD) was calculated using the equation:

(3) $$MD=SEM*1.96*\sqrt{2}$$

to examine the individual differences for each variable from test 1 to test 2. The coefficient of variation (CoV) was also calculated to display a normalized measure of the SEM using the equation:

(4) $$CoV=\frac{SEM}{GrandMean}*100.$$

Based on previous recommendations [38], a CoV of <10% was used as an indication of sufficient absolute reliability. However, the overall reliability of the measures was characterized by taking into account the ICC value, in conjunction with the CoV, SEM, and the MD. The effect size for each variable of the ANOVAs was expressed as the partial eta squared (pn^2^). An a priori alpha level was set at 0.05, and all of the data were analyzed using IBM SPSS Statistical Software Version 28 (IBM SPSS Inc., Chicago, IL, USA).

## 3. Results

**Figure 1:**
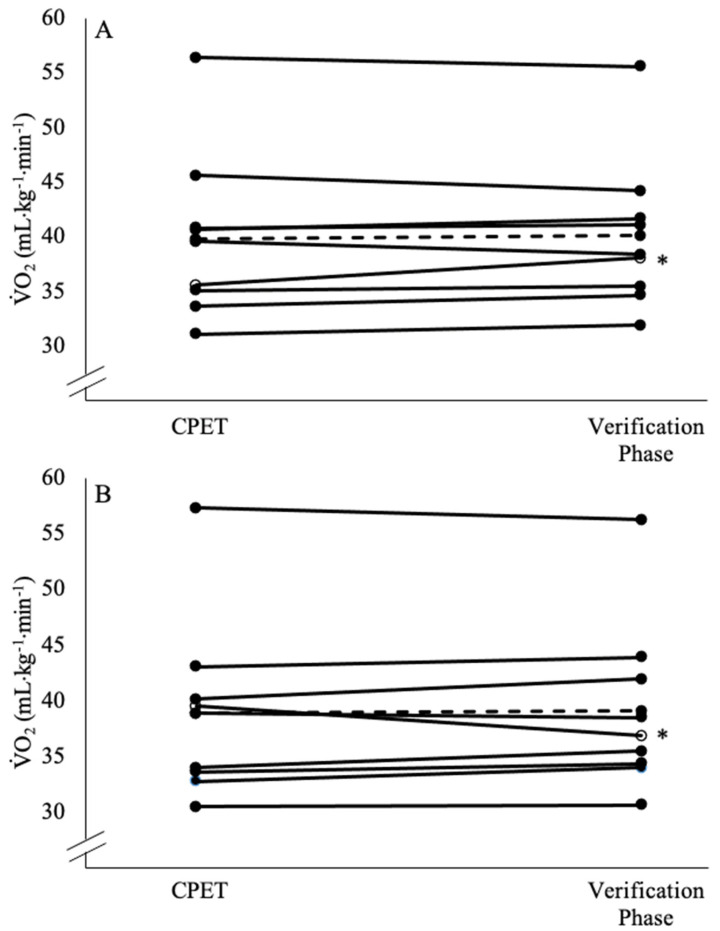
Individual (solid line and closed circles) and mean (dashed line) $\dot{V}$O_2_ responses from the cardiopulmonary exercise test (CPET) and the verification phase for test 1 (**A**) and test 2 (**B**). * indicates an individual (solid line open circles) exceeded the minimal difference to be considered real (2.14 mL·kg^−1^·min^−1^) between the CPET and verification phase.

**Figure 2:**
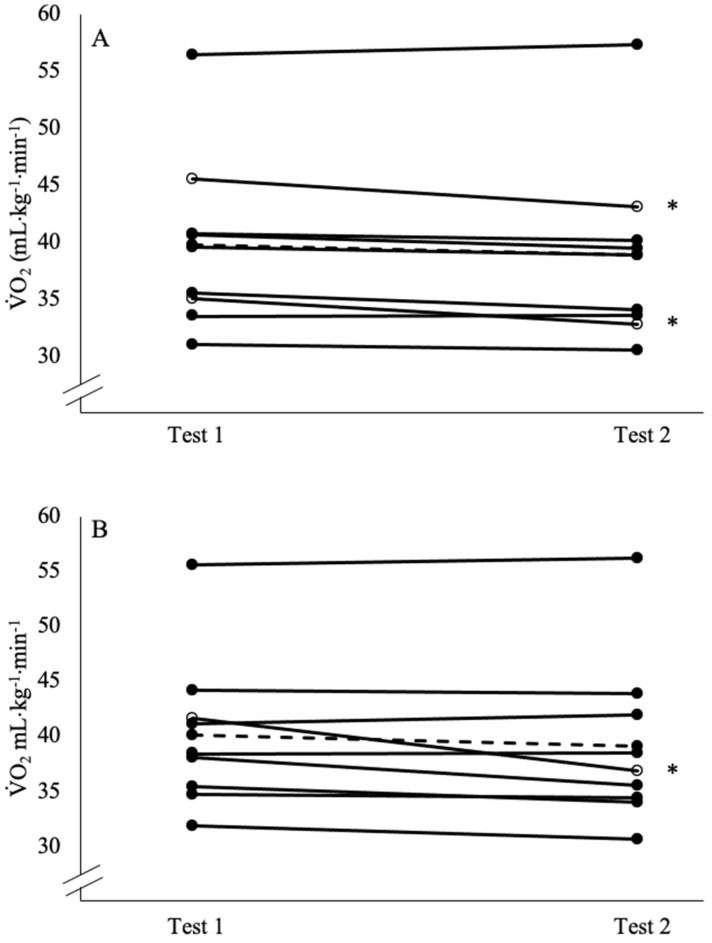
Individual (solid line and closed circles) and mean (dashed line) $\dot{V}$O_2_ responses from the cardiopulmonary exercise test (CPET) 1 compared to test 2 (**A**), and the verification phase test 1 compared to test 2 (**B**). * indicates an individual (solid line and open circles) exceeded the minimal difference to be considered real for the CPET (2.14 mL·kg^−1^·min^−1^) or the verification phase (3.52 mL·kg^−1^·min^−1^).

The individual responses and the mean ± SD for each variable (test 1 $\dot{V}$O_2CPET_, test 2 $\dot{V}$O_2CPET_, test 1 $\dot{V}$O_2verification_, and test 2 $\dot{V}$O_2verification_) are listed in Table 1 and shown in Figure 1 and Figure 2. The results of the two-way repeated measures ANOVA for peak $\dot{V}$O_2_ demonstrated no significant test x method interaction (*F* = 0.018, *p* = 0.896, pη^2^ = 0.002) and no main effect for the method (*F* = 0.605, *p* = 0.459, pη^2^ = 0.070), but there was a significant main effect for the test (*F* = 8.465, *p* = 0.043, pη^2^ = 0.419). Followup comparisons indicated that collapsed across the method (i.e., the average of both the CPET and verification phase), test 1 (39.2 ± 7.2 mL·kg^−1^·min^−1^) was significantly higher than test 2 (38.3 ± 7.7 mL·kg^−1^·min^−1^). The mean ± SD T_Lim_ for test 1 and test 2 for the CPET and verification phase, as well as the peak power output (PPO) from the CPET, the verification phase power output, and the maximal HR, RER, and RPE for the CPET and verification phase are listed in Table 2. There were no significant interactions, but there was a main effect for the method that indicated the T_Lim_ for the CPET was longer than the verification phase (*p* < 0.001), and the peak power output (*p* < 0.001) and RER (*p* < 0.001) during the CPET were greater than the verification phase. There were no interactions or main effects for the test or method for the maximal HR or RPE (*p* = 0.062–0.512).

The reliability statistics are presented in Table 3. The test 1 $\dot{V}$O_2CPET_ was significantly higher than the test 2 $\dot{V}$O_2CPET_ (*F* = 6.563, *p* = 0.034, pη^2^ = 0.451), but there was no difference between the test 1 $\dot{V}$O_2verification_ and the test 2 (*F* = 2.833, *p* = 0.131, pη^2^ = 0.261). The ICC values of the $\dot{V}$O_2CPET_ (R = 0.984) and the $\dot{V}$O_2verification_ (R = 0.964) indicated both methods demonstrated ‘excellent’ test–retest reliabilities [37]. The CoV for the $\dot{V}$O_2CPET_ (1.98%) and $\dot{V}$O_2verification_ (3.30%) were both below the 10% threshold used to be considered reliable [38]. Two subjects exceeded the MD (2.14 mL·kg^−1^·min^−1^; 0.12 L·min^−1^) for the $\dot{V}$O_2CPET_ test–retest. In addition, one subject exceeded the MD (3.30 mL·kg^−1^·min^−1^; 0.22 L·min^−1^) for the $\dot{V}$O_2verification_ test–retest. Lastly, one subject demonstrated a $\dot{V}$O_2CPET_ that was greater than the $\dot{V}$O_2verification_ by a value that exceeded the MD (2.14 mL·kg^−1^·min^−1^), and one subject demonstrated a $\dot{V}$O_2verification_ that was greater than the $\dot{V}$O_2CPET_ by a value that was greater than the MD (Table 1).

## 4. Discussion

The purpose of this study was to use a test–retest approach to examine the reliability, mean, and individual differences between the $\dot{V}$O_2CPET_ and $\dot{V}$O_2verification_ in healthy, recreationally trained, and well-motivated women during cycle ergometry. The recommendation for researchers to perform verification phase testing when determining the $\dot{V}$O_2max_ in all populations has become more prevalent [18]. It is of note that the verification phase may be necessary in novice, unmotivated, older, or especially diseased populations [39]. However, it has been suggested that the verification phase may not be necessary in young healthy subjects that are accustomed to exhaustive exercise [18]. In addition, previous studies in this population have demonstrated highly reproducible $\dot{V}$O_2max_ values based on the group mean responses [18,23,27,28,29]. The findings of the current study provide additional support to this notion as both the $\dot{V}$O_2CPET_ and $\dot{V}$O_2verification_ demonstrated “excellent” reliabilities based on the ICC along with the MD, SEM, and CoV (Table 3). Although the test–retest mean responses for the $\dot{V}$O_2CPET_ indicated systematic variability (test 1 > test 2), this mean difference reflected a real difference, based on the MD, for only two of the nine subjects. The use of several indices of reliability allows for the determination of the absolute and relative reliability, which enables an individual to compare across studies [17,40]. The ICC, MD, SEM, and CoV for the $\dot{V}$O_2CPET_ and $\dot{V}$O_2verification_ in this study were consistent with previous work examining the CPET and verification phase protocols (ICCs = 0.89–0.99, MDs = 0.17–0.21 L⋅min^−1^, SEMs = 0.06–0.16 L⋅min^−1^, and CoVs = 2.1–3.8%) [6,11,12,27,28,29]. Thus, the current findings further supported that the $\dot{V}$O_2CPET_ and $\dot{V}$O_2verification_ can be reliably determined in younger, healthy, and well-motivated subjects.

Previous work investigating the need for a verification phase in the determination of the $\dot{V}$O_2max_ has examined the mean responses of the $\dot{V}$O_2_ determined from the CPET compared to the $\dot{V}$O_2_ determined from the verification phase. Other works have demonstrated no mean difference in the $\dot{V}$O_2max_ between the CPET and verification phase, yet still recommend its use in all populations [8,25]. However, as has been previously pointed out [23,24], the examination of the individual responses is more important than the mean responses in regard to the attainment of the highest $\dot{V}$O_2_ ($\dot{V}$O_2max_). In the current study, there was no main effect for method (i.e., no difference for the $\dot{V}$O_2_ determined from the CPET vs. the verification phase). This lack of difference between the CPET and the verification phase is consistent with previous studies [4,6,7,8,11] but is also in contrast to other works [27,28,29,41] that demonstrated that the CPET $\dot{V}$O_2max_ was significantly greater than the verification phase. However, in contrast to other works [4,6,7,8,11,12,27,28,29], the $\dot{V}$O_2_ from test 1 in this study was significantly greater than test 2, collapsed across the CPET and verification phase. It is important to note that the mean difference between test 1 (39.2 ± 7.2 mL·kg^−1^·min^−1^) and test 2 (38.3 ± 7.7 mL·kg^−1^·min^−1^) was 0.9 ± 1.2 mL·kg^−1^·min^−1^ (~2.2%), and there were no differences in the time to exhaustion, power output, or maximal HR between test 1 and test 2. On an individual basis, only two of the nine subjects for the CPET and one of the nine subjects for the verification phase test–retest exceeded the MD for the test–retest responses. These findings demonstrate a potential pitfall of using the mean responses alone to evaluate the changes in the $\dot{V}$O_2max_ across time or as the result of a training or dietary intervention. That is, evaluation of the mean response alone would suggest a significant change across time; however, this reflected a real difference for only three out eighteen total test–retest responses. Thus, these findings illustrate that using the mean responses may not be sufficient to fully examine the proper methodology for the measurement of the $\dot{V}$O_2max_ and highlight the potential usefulness in examining changes across time and the need for the examination of individual responses.

The utility of the verification phase is to determine on an individual basis whether the $\dot{V}$O_2_ obtained from the CPET is truly the maximal $\dot{V}$O_2_ that an individual is capable of producing. However, in past works, the threshold that has been used to determine whether there were individual differences was set at 2–3% between measures [4,7,12,14]. Using this threshold presents a flawed approach as it does not consider the standard error of the measurement of the $\dot{V}$O_2_ in addition to the biological variability associated with the measurement. The use of the minimal difference (MD) to be considered real provides a threshold with increased statistical backing to determine whether the differences between the CPET and the verification phase are real differences or are just due to the error of the measure [17]. The MD for the measurement of the $\dot{V}$O_2_ from the CPET and verification phase has previously been quantified in men and women during treadmill running [27,28] and in men during cycle ergometry [29], but it has yet to be determined for women during cycle ergometry. Therefore, the quantification of the MD in women during cycle ergometry (2.14 mL·kg^−1^·min^−1^) may allow future researchers to examine the individual responses in the $\dot{V}$O_2max_ to potential changes in interventional studies. Using this approach, one individual demonstrated a $\dot{V}$O_2verification_ that exceeded the $\dot{V}$O_2CPET_ by more than the MD, while one individual demonstrated a $\dot{V}$O_2CPET_ that was greater than the $\dot{V}$O_2verification_ by more than the MD. These data suggest that a verification phase is not necessary in the measurement of the $\dot{V}$O_2max_ in healthy well-motivated women on a cycle ergometer. In addition, the MD may provide a valuable tool to examine individual differences in the $\dot{V}$O_2max_ across time or as the result of an intervention.

### Limitations

The variation in the $\dot{V}$O_2max_ for those subjects who exceeded the MD for the test–retest responses may be a result of the increased variation in the $\dot{V}$O_2_ due to biological factors and may highlight ‘responders’ vs. ‘non-responders’ [42] due to factors that may influence the maximal performance, such as the time of day [43] or the menstrual cycle phase [44], in addition to other factors such as diet, hydration, or sleep [42]. However, additional work is needed to explore the magnitude of the effect that these factors may have on individual performance measures. While the time of day of testing was kept consistent in the current study (±2 h), Knaier et al. [43] demonstrated that individual $\dot{V}$O_2_ values can vary even when there are minor variations (<3 h) in the time of day that the testing is repeated. Furthermore, Lebrun et al. [44] demonstrated that the $\dot{V}$O_2max_ can vary across the menstrual cycle phases. The individual menstrual cycle phase was not tracked in the current study; however, all test–retest responses were recorded within 48–72 h. Although it is possible that menstrual cycle phase transitions introduced sufficient variability to alter the $\dot{V}$O_2max_, based on the timing of the testing protocol and the lack of effect for these same subjects on the verification phase $\dot{V}$O_2_ responses, this seems unlikely to be the primary driver of the variations in the $\dot{V}$O_2max_. Future researchers should examine factors that may impact maximal day-to-day performance to determine the possible magnitude of these effects. In addition, future studies should quantify the MD using a larger sample size. 

## 5. Conclusions

The results of the current study suggested that its use in healthy well-motivated women who are accustomed to maximal exhaustive exercise may not be necessary. The performance of additional maximal tests increases the demand on subjects to push themselves to their limit more than may be necessary and increases the burden on researchers to perform these additional tests. Day et al. [30], and more recently Poole and Jones [18] have previously made this claim; however, there were no specific data as support. The current study has shown that there were no mean differences between the $\dot{V}$O_2CPET_ and $\dot{V}$O_2verification_, and both measures demonstrated ‘excellent’ test–retest reliabilities. Therefore, these data support the claims made in previous work [18,30], which suggested young, healthy, and well-motivated subjects may not need to perform additional tests in the measurement of the $\dot{V}$O_2max_. In addition, this study has added to the quantification of the MD that has previously been determined for men in running and cycling and in women in running but had not been derived for women in cycling. There were two individuals who demonstrated differences in the $\dot{V}$O_2CPET_ test–retest and one individual who demonstrated differences in the $\dot{V}$O_2verification_ test–retest. Thus, the few individual differences in combination with the lack of mean difference between the CPET and verification phase responses, suggested that the verification phase may not be necessary in healthy motivated women. The MD allows for the examination of individual responses with a threshold that is based on the standard error of the measure and presents an improvement on the 2–3% threshold, which has previously been used. Thus, the MD may prove useful for other researchers to examine the individual $\dot{V}$O_2max_ responses to potential training or nutritional intervention studies.

## Figures and Tables

**Table 1:** Mean ± SD and individual responses for the highest $\dot{V}$O_2_ values from the cardiopulmonary exercise test ($\dot{V}$O_2CPET_) and the verification phase ($\dot{V}$O_2erification_) for test 1 (T1) and test 2 (T2).

Subject	$T1\;\dot{V}$O_2CPET_	$T2\;\dot{V}$O_2CPET_	$T1\;\dot{V}$O_2verification_	$T2\;\dot{V}$O_2verification_
1	45.5 *	43.1	44.1	43.9
2	31.0	30.5	31.9	30.6
3	40.6	39.5 ^‡^	41.7 ^†^	36.8
4	40.8	40.1	41.1	41.9
5	35.1 *	32.8	35.4	34.0
6	35.6	34.0	38.0 ^‡^	35.4
7	56.4	57.3	55.5	56.2
8	33.6	33.6	34.6	34.3
9	39.5	38.8	38.4	38.5
**Mean**	**39.8**	**38.9**	**40.1**	**39.1**
**SD**	**7.6**	**8.1**	**6.9**	**7.6**

* indicates test 1 versus test 2 for the CPET exceeded the minimal difference to be considered real (MD) (2.14 mL·kg^−1^·min^−1^). ^†^ indicates test 1 versus test 2 for the verification phase exceeded the MD (3.52 mL·kg^−1^·min^−1^). ^‡^ indicates the CPET versus the verification phase exceeded the MD (2.14 mL·kg^−1^·min^−1^).

**Table 2:** Mean ± SD for time to exhaustion (T_Lim_), peak power output, heart rate (HR), respiratory exchange ratio (RER), and rating of perceived exertion (RPE) for test 1 (T1) and test 2 (T2) cardiopulmonary exercise test (CPET) and verification phase.

	T1 CPET	T2 CPET	T1 Verification	T2 Verification
T_Lim_ (min) *	11.25 ± 1.27	11.43 ± 1.23	3.01 ± 0.98	3.14 ± 0.88
Power Output (W) *	203 ± 23	203 ± 23	183 ± 21	183 ± 21
HR (b·min^−1^)	182 ± 6	180 ± 8	181 ± 8	180 ± 8
RER *	1.18 ± 0.05	1.20 ± 0.06	1.08 ± 0.07	1.11 ± 0.05
RPE	19 ± 1	19 ± 1	19 ± 1	19 ± 1

Power output for the CPET represents the peak power output (PPO), and for the verification phase represents 90% of the peak power output from the CPET. * T_Lim_, power output, and RER during the CPET was greater than verification phase, collapsed across test (*p* < 0.001).

**Table 3:** Reliability analyses including the mean ± SD, intraclass correlation coefficient (ICC), standard error of the measure (SEM), minimal difference (MD) to be considered real, and the coefficient of variation (CoV) for the $\dot{V}$O_2_ from the cardiopulmonary exercise test ($\dot{V}$O_2CPET_) and the verification phase ($\dot{V}$O_2verification_).

$\dot{V}$O_2_ (Mean ± SD)	Test 1	Test 2	*p*	ICC (95% CI)	SEM (mL·kg^−1^·min^−1^)	MD (mL·kg^−1^·min^−1^)	CoV (%)
$\dot{V}$O_2CPET_	39.8 ± 7.6	38.9 ± 8.1	0.034	0.984 (0.879–0.997)	0.77	2.14	1.98
$\dot{V}$O_2verification_	40.1 ± 6.9	39.1 ± 7.6	0.131	0.964 (0.841–0.992)	1.27	3.53	3.30

## Data Availability

The data presented in this study are available upon request to the corresponding author.
